# Genome-Wide Identification and Characterization of the NAC Gene Family and Its Involvement in Cold Response in *Dendrobium officinale*

**DOI:** 10.3390/plants12203626

**Published:** 2023-10-20

**Authors:** Qianyu Yang, Zhihui Li, Xiao Wang, Chunqian Jiang, Feihong Liu, Yuxin Nian, Xiaoyun Fu, Guangzhu Zhou, Lei Liu, Hui Wang

**Affiliations:** 1 College of Forestry, Shenyang Agricultural University, Shenhe District, Shenyang 110866, China; yangdayu0055@163.com (Q.Y.); wx1970218@163.com (X.W.); lfh-303@163.com (F.L.); 2004500017@syau.edu.cn (Y.N.);; 2 Institute of Applied Ecology, Chinese Academy of Sciences, Shenyang 110016, China; 3 Key Laboratory of Tree Breeding and Cultivation of National Forestry and Grassland Administration, Research Institute of Forestry, Chinese Academy of Forestry, Beijing 100091, Chinaliulei519@caf.ac.cn (L.L.)

**Keywords:** cold stress, *Dendrobium officinale*, expression profiles, NAC gene family

## Abstract

The NAC (NAM, ATAF1/2 and CUC2) gene family is one of the largest plant-specific transcription factor families, functioning as crucial regulators in diverse biological processes such as plant growth and development as well as biotic and abiotic stress responses. Although it has been widely characterized in many plants, the significance of the NAC family in *Dendrobium officinale* remained elusive up to now. In this study, a genome-wide search method was conducted to identify NAC genes in *Dendrobium officinale* (DoNACs) and a total of 110 putative DoNACs were obtained. Phylogenetic analysis classified them into 15 subfamilies according to the nomenclature in Arabidopsis and rice. The members in the subfamilies shared more similar gene structures and conversed protein domain compositions. Furthermore, the expression profiles of these DoNACs were investigated in diverse tissues and under cold stress by RNA-seq data. Then, a total of five up-regulated and five down-regulated, cold-responsive DoNACs were validated through QRT-PCR analysis, demonstrating they were involved in regulating cold stress response. Additionally, the subcellular localization of two down-regulated candidates (DoNAC39 and DoNAC58) was demonstrated to be localized in the nuclei. This study reported the genomic organization, protein domain compositions and expression patterns of the NAC family in *Dendrobium officinale*, which provided targets for further functional studies of DoNACs and also contributed to the dissection of the role of NAC in regulating cold tolerance in *Dendrobium officinale*.

## 1. Introduction

The NAC gene family is one of the largest plant-specific transcription factor families, playing a crucial role in regulating growth and development as well as responses to diverse biotic and abiotic stresses in plants [1,2]. The NAC family is named according to three conserved domains, including petunia no apical meristem (NAM), ATAF1/2 and cup-shaped cotyledon (CUC) [3,4]. The NAM gene was firstly cloned and functionally characterized in petunias, which was found to determine the position of the shoot apical meristem and primordia or in plants [5]. Subsequently, the ATAF1/2 and CUC genes were successively found and identified [6]. Generally, NAC proteins have a typical and conserved N-terminal region (NAC domain), together with a variable C terminal [7]. The NAC domain is about 150 AA in length, comprising five subdomains (A–E), of which subdomains A, C and D are rather conserved, while subdomains B and E are highly viable with special functions [1,8,9,10]. At the same time, the C-terminal region is mainly involved in transcriptional activation or repression to achieve its regulation function (TAR or TRR) [11,12].

Extensive studies have demonstrated that NAC transcription factors are widely involved in various biological processes, such as cell division, shoot apical meristem, flower development and fruit ripening, as well as leaf senescence and so on [12,13,14]. *NAC29* and *NAC21* were reported to play an indispensable role in cellulose synthesis in rice [15]. Over-expression of *OsNAC2* promoted shoot branching and also increased the growth of rice tiller buds [16]. In Arabidopsis, *ANAC046* and *ANAC087* regulated the programmed cell death of lateral roots [17] and *NAP* functioned as an important regulator to control leaf senescence and floral morphogenesis [18,19]. Meanwhile, the NAC family has also been found to participate in the response to diverse stresses [13,20]. It is reported that *SNAC2* functioned to regulate cold and salt tolerance in rice [21]. Over-expression of *ONAC022* significantly improved drought and salt tolerance in rice [22]. In tomatoes, the NAC protein JUNGBRUNNEN1 can enhance drought tolerance by activating the expression of stress-induced genes [23]. In Arabidopsis, *ANAC013* and *ANAC017* regulated the oxidative stress response by mediating mitochondrial feedback signals [24,25]. *MaNAC1* interacted with *MaCBF1* to modulate cold tolerance in bananas [26]. In light of its importance, the NAC gene family has been systematically investigated in many plant species at the whole-genome level, including Arabidopsis [1], rice [1], maize [27], soybeans [28] and wild emmer wheat [29], some of which have been well characterized functionally [30].

*Dendrobium officinale* is a well-known medicinal and ornamental herb belonging to the orchid family Dendrobium genus, which originated from East Asia and Southeast Asia, mainly in China, Japan, India and other regions [31]. *D. officinale* contains rich bioactive components such as polysaccharides, dendrobium alkaloids, flavonoids and so on, and is used as a precious Chinese herbal medicine with a medicinal-use history of more than 2000 years [32]. Due to its rareness and significant medicinal value, the production of *D. officinale* not only provides the necessary plant material for traditional Chinese medicine, but also brings huge economic benefits to accelerate rural revitalization. However, abiotic stresses, especially cold stress, can severely impair the production of *Dendrobium officinale*, which reduces the yield of *D. officinale* and also damages the medicinal value [33]. Thus, it is urgent to breed for cold-tolerant *D. officinale* by making use of the elite genes associated with cold tolerance. Although previous studies have revealed that NAC genes played crucial roles in controlling cold tolerance in different plants [12,34], the significance of NAC in *D. officinale* remained elusive up to now. In this study, we performed a genome-wide search of the NAC family in *D. officinale* at the genome scale and the phylogenetic relationship and a conserved motif of the putative DoNACs were investigated. Then, their expression patterns were detected in diverse tissues and under cold stress by RNA-seq data, and 10 cold-responsive candidates were further validated by QPCR analysis. It is the first study to identify the NAC gene family in *D. officinale*, which provides potential targets for further functional study and contributes to improving the cold tolerance in *D. officinale* and beyond.

## 2. Results

### 2.1. Identification of the NAC Gene Family in D. officinale

To globally obtain the NAC genes in the *D. officinale* genome, a genome-wide search method was conducted using both HMMSearch and BLASTP methods. After removing redundant sequences and confirming the conserved NAC or NAM domains, a total of 110 putative NAC genes were identified and designated as *DoNAC1* to *DoNAC110* (Table 1). All of them contained a conserved NAC domain (PF01849) or NAM domain (PF02365), indicating the accuracy of the prediction. The protein length of these *DoNACs* ranged from 97 (*DoNAC47*) to 1174 AA (*DoNAC13*) with an average length of 292 AA. Their molecular weight (MW) ranged from 11.14 (*DoNAC47*) to 129.50 *(DoNAC13*) kDa with an average of 33.20 kDa and the pI value ranged from 4.19 (*DoNAC93*) to 10.28 (*DoNAC3*) with an average of 7.29. These diverge protein characteristics suggested their divergent functions. Finally, their subcellular localization was predicted and results showed that most of the DoNACs were located in the nucleus, although some of them were predicted into cytoplasm, indicating that DoNAC acted as transcription factors to play regulatory roles in biological processes in *D. officinale* (Table 1).

### 2.2. Phylogenetic and Conserved Motif Analysis of DoNACs

**Figure 1:**
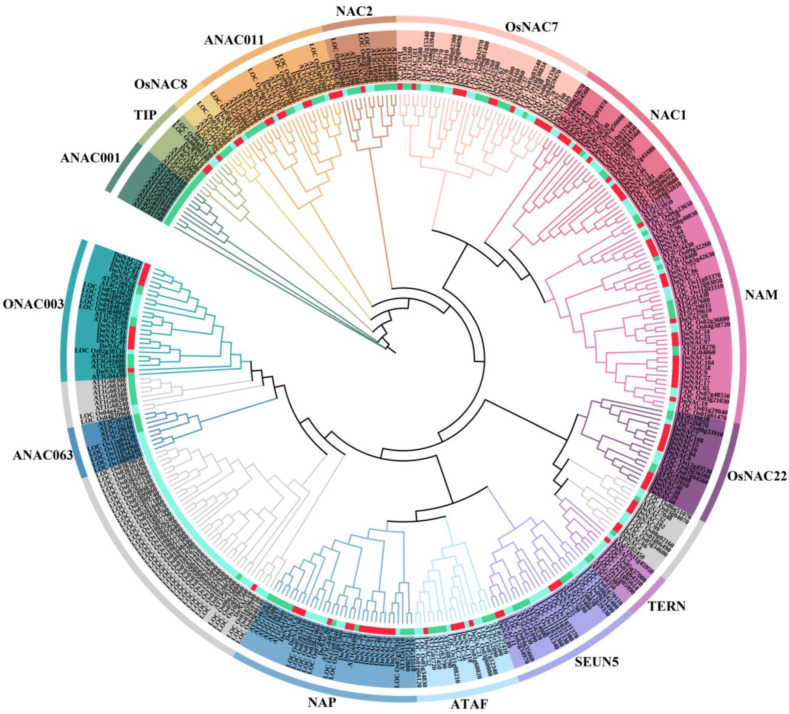
Phylogenetic analysis of the NAC proteins of Arabidopsis, rice and *D. officinale*. The phylogenetic tree was constructed using the neighbor-joining (NJ) method with 1000 bootstrap replications.

To understand the phylogenetic relationships of these putative *DoNAC*s, the identified 110 DoNACs, together with 105 Arabidopsis and 137 rice NAC proteins, were aligmented to perform phylogenetic analysis. Based on their phylogenetic relationships, these NAC proteins could be divided into 15 groups; homologous pairs were also determined, having a closer phylogenetic relationship to cluster together (Figure 1). According to the classification criteria of Arabidopsis and rice [1], these DoNAC proteins categorized into different subfamilies, providing some clues about the function of these DoNACs. It was shown that the DoNACs were present in all of the 15 subfamilies, suggesting that no obvious NAC gene loss occurred in *D. officinale*. Furthermore, the number of DoNACs in each subfamily ranged from two to 19, of which the NAM subfamily possessed the most members while NAC2, TIPANAC001 and OsNAC8 contained only two DoNACs. Meanwhile, it is obvious that several NAC proteins from the same species generally cluster together, which might be the result of the segmental duplication events of the NAC genes in their genomes [29]. Compared to *D. officinale* and Arabidopsis, duplication events are more frequent in rice.

**Figure 2:**
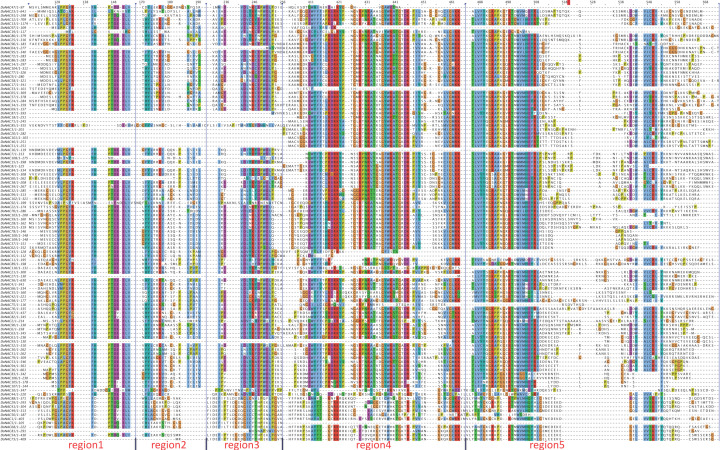
The conserved region identified in the NAC protein in *D. officinale* through multi-sequence alignment.

**Figure 3:**
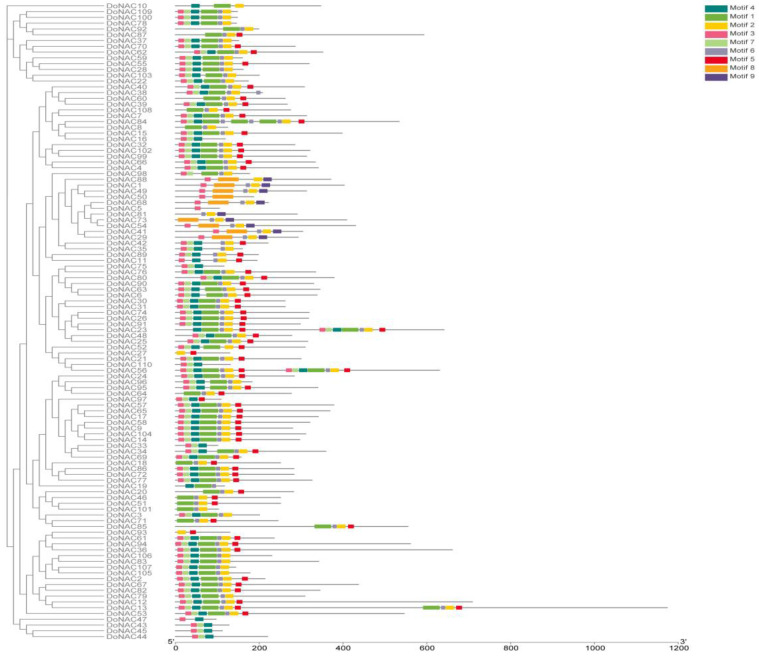
The conserved domain organization in the NAC protein in *D. officinale*.

Based on the multi-sequence alignment, the conserved region in these 110 DoNAC proteins was further identified (Figure 2). Results showed that the N-terminus seemed rather conservative among them, while the C-terminus was highly variable, which was consistent with previous studies. A total of five conserved regions were found, four of which were located at the N-terminus, and the remaining one was adjacent to the C-terminus. It was found that protein conservation gradually becomes lower from N to C terminus. Furthermore, the conserved functional domain among them were analyzed using the MEME tool to obtain clues about the function that they might be involved in. In total, nine highly conserved functional domains were found and all of them were found adjacent to the N-terminus, with none in found in the C-terminus (Figure 3). Most of the DoNACs possessed Motif 1 to 8, suggesting they are highly conserved in the functional domain composition. It is no accident that all of the DoNAC proteins contained the NAM, ATAF and CUC domains.

At the same time, Motif 9 was only found in a few DoNACs, which have relatively high protein sequence divergents in conserved protein regions two, three and four, indicating that Motif 9 might be the novel formed domain with some specific function. Further analysis found that Motif 9 is associated with proto2021_ 04 Eukaryote PS00880, namely the Acyl CoA binding (ACB) domain. These NAC proteins, having Motif 9, may participate in the regulation of energy metabolism. Finally, it is obvious that members in the same subfamily have similar conserved protein regions and motif compositions, suggesting their similar biological function.

### 2.3. Cis-Element Analysis of DoNACs

**Figure 4:**
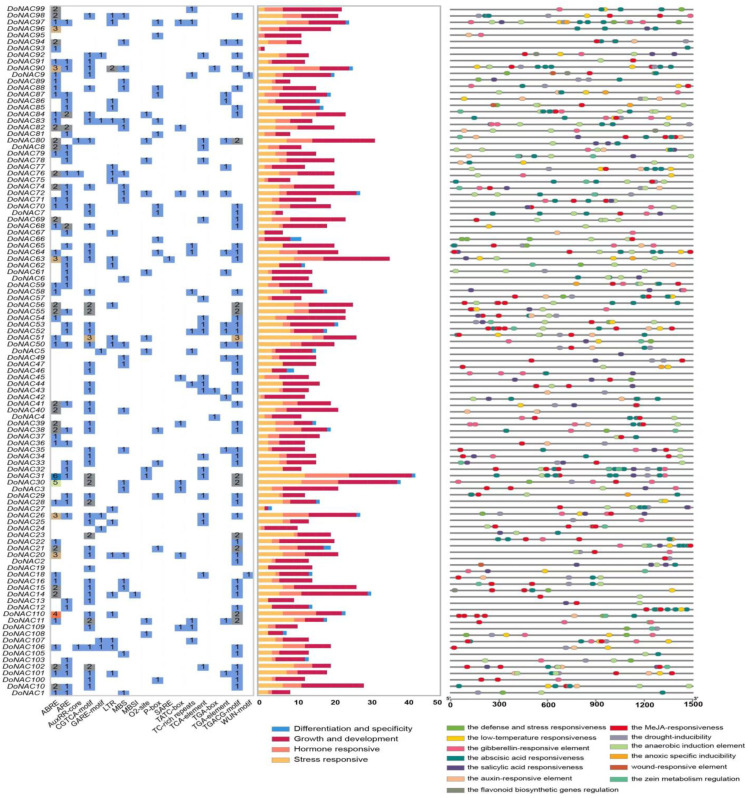
The cis-element composition and function categories in the promoter regions of DoNACs.

The 1.5 kb genomic sequences upstream from the transcription start sites (TSS) of these DoNACs were extracted to predict the cis-acting elements. Totally, 1890 cis-elements were found in them with an average of 17.2 elements per *DoNAC* gene, which are widely associated with growth and development (1004), differentiation and specificity (33) as well as hormone responsiveness (299) and stress responsiveness (562) (Figure 4 and Table S1). A large number of cis-elements associated with growth and development were found in the *DoNAC*s, such as CAT-box (related to meristem expression), circadian (related to circadian control), GCN4_motif (related to endosperm expression) and MSA-like (related to cell cycle regulation). Almost each of the *DoNAC*s contained the cis-element are involved in light responsiveness, including G-box, Sp1, TCCC-motif and ATCT-motif. HD-Zip I elements that are involved in the differentiation of the palisade mesophyll cells, were found in nine *DoNAC*s, and an RY-element that is involved in seed-specific regulation was also found in nine *DoNAC*s, suggesting that *DoNAC*s have a function in tissue differentiation and specificity. Particularly, the promoter region of *DoNAC66* possessed three RY-elements, indicating it might play an important role in flower or seed morphogenesis. Furthermore, the cis-elements related to diverse hormones were also widely identified in 93 *DoNAC*s, including abscisic acid (ABRE), auxin (AuxRR-core and TGA-box) and gibberellin (P-box and TATC-box), of which DoNAC31 contained 11 ABRE elements, *DoNAC3* contained two TATC-box and *DoNAC101* had two TGA-box. Finally, 105 *DoNAC*s (95.5%) were found to have 562 stress-responsive cis-elements, indicating their indispensable role in stress response in *D. officinale*. In detail, 46 *DoNA*Cs had MBS elements (involved in drought-inducibility) and 36 *DoNAC*s had LTR elements (involved in low-temperature responsiveness). Among them, *DoNAC90* had three LTR elements and *DoNAC56* and *DoNAC76* had two LTR elements, suggesting they might be involved in cold stress response.

### 2.4. Expression Profile of DoNACs in Different Tissues

**Figure 5:**
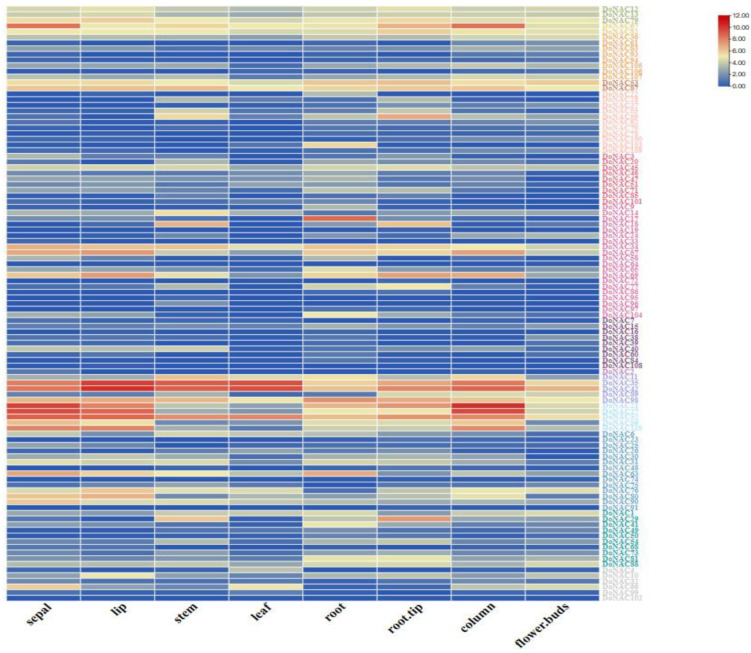
Expression patterns of DoNACs in 8 different tissues.

The expression profiles of these *DoNAC*s were investigated in eight different tissues based on the public RNA-seq data (PRJNA348403) (Figure 5 and Table S2). Results showed that 105 out of 110 *DoNAC*s were expressed in at least one of the tissues. Based on their expression profiles, they could be clustered into 10 groups, including eight tissue-specific groups, one group showing expression in all tissues and one group with no obvious expression tendency. We found that 20 DoNACs displayed continuously high expression in all of the tested tissues, including *DoNAC35*, *DoNAC42*, *DoNAC52*, *DoNAC53*, *DoNAC87*, *DoNAC98* and so on, which might function as crucial regulators associated with the growth and development of *D. officinale*. Meanwhile, tissue-specific DoNACs were also identified. *DoNAC66* showed significant high expression in sepal, lip, leaf and flower buds, which was consistent with it having three RY-elements in the promoter region. DoNAC17, DoNAC22 and DoNAC103 displayed highest expression in root tissue compared to other tissues, indicating their important role in root development. DoNAC26 showed high expression in leaf tissue, and *DoNAC28* was found to be highly expressed in stem and root tip tissues and *DoNAC100* was highly expressed in both column tissue and flower buds. *DoNAC108* was found to be lip—specific. These tissue-specific DoNACs provided the potential target for further functional study to reveal their regulation function.

### 2.5. Expression Analysis of DoNACs under Cold Stress

**Figure 6:**
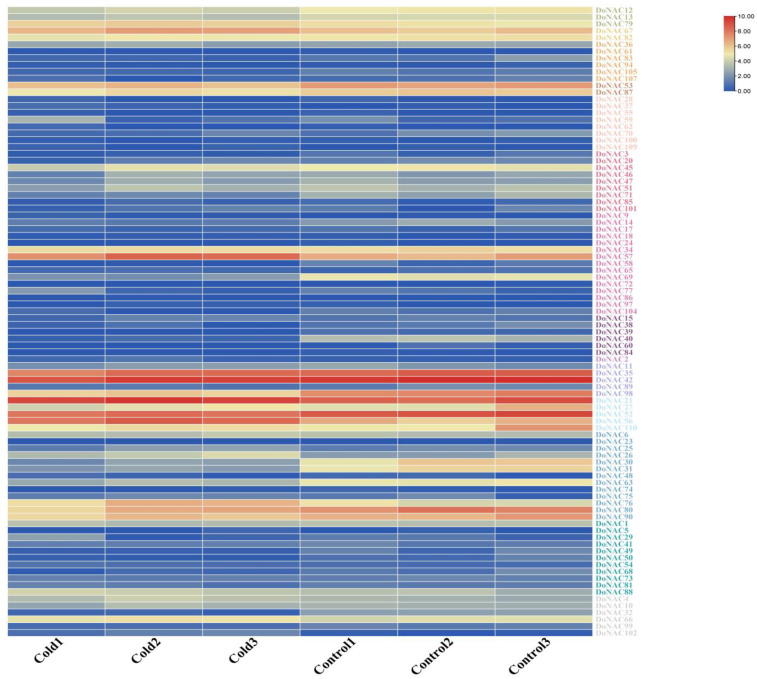
Expression patterns of DoNACs under cold stress. Cold and control represent under cold and control treatment. Three biological replications were used.

To identify the candidate DoNACs involved in response to cold stress, we further investigated their expression patterns under cold treatment (Figure 6 and Table S3). The results showed that a total of 87 out of 110 DoNACs showed expression under cold treatment, proving the crucial role of NAC in cold response. Compared to the control, most of the DoNACs showed lower expression levels under cold stress and the differentially expressed DoNACs between them were detected. In total, 31 DoNACs were found to be differentially expressed between the cold and control treatments, which could be considered cold-responsive DoNACs. Among them, 11 were up-regulated and 20 were down-regulated, respectively. The expression level of *DoNAC102* showed 9.8-fold higher under cold stress than that of control, which is the most significantly up-regulated expression gene, followed by *DoNAC56, DoNAC57* and *DoNAC26*. While *DoNAC58* and *DoNAC39* were the most significantly down-regulated expression genes, which displayed 42 and 37 times lower expression under cold stress compared to CK. These cold-responsive DoNACs could be used as important candidates for revealing their regulatory function in the cold stress response and tolerance in *D. officinale*.

### 2.6. Validation of the Cold-Responsive DoNACs by qRT-PCR Analysis and Subcellular Localization

**Figure 7:**
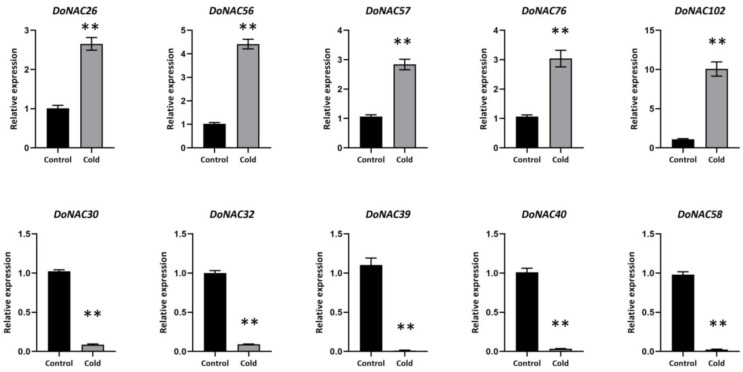
The expression levels of 10 randomly selected cold-responsive DoNACs under control and cold stress by qRT-PCR methods. ** represents significant level at 0.01.

To explore the key NAC gene underlying cold response, five up-regulated (*DoNAC26, DoNAC56, DoNAC57, DoNAC76* and *DoNAC102*) and five down-regulated (*DoNAC30, DoNAC32, DoNAC39, DoNAC40* and *DoNAC58*) candidates were randomly selected based on the RNA-seq analysis to verify their expression by qRT-PCR analysis (Figure 7). The results showed that the expression based on the qRT-PCR method was completely consistent with that of RNA-seq analysis. The *DoNAC102* was validated to be the most significantly up-regulated gene under cold stress, which was induced by cold to more than 10 times higher compared to CK. *DoNAC26, DoNAC56, DoNAC57* and *DoNAC76* were also induced to up-regulated expression by cold with different levels. It is interesting that all five down-regulated genes were validated to be induced to low expression by cold and their expression levels were rather low or seemed to show no expression. These validated cold-responsive DoNACs could be considered the key candidates underlying cold stress response, which are useful targets for further studies to reveal their function and role in cold adaptation and tolerance in *D. officinale*, especially the identified novel down-regulated candidates, which provided the valuable target to improve cold tolerance through a genome-editing approach [35].

**Figure 8:**
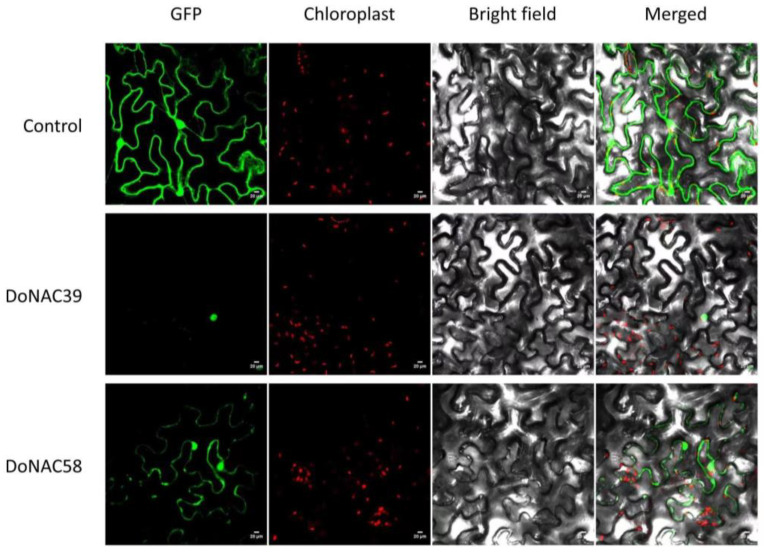
Subcellular localization of 2 recombinant CaMV35S: DoNAC-GFP-fused proteins transiently expressed in tobacco leaf cells.

Furthermore, the subcellular localization of two down-regulated candidates (*DoNAC39* and *DoNAC58*) was investigated. OsNAC-GFP fusion protein transient vectors were constructed and then injected into tobacco leaf cells (Figure 8). The results showed that both of them were localized in the nucleus, demonstrating their role as transcription factors to regulate downstream biological processes.

## 3. Discussion

*Dendrobium officinale* is a rare and precious herb with huge ornamental, medicinal and cultural value which has been used as an ingredient in traditional medicine for thousands of years in China and is also widely considered a medicinal material in many other Asian countries [31]. Recently, a large number of studies have reported that *D. officinale* is rich in bio-active compounds, including polysaccharides, flavonoids, alkaloids and multiple amino acids, which have been demonstrated to have anti-oxidation, immune regulation and anti-cancer effects [36,37]. With the shortage of wild *D. officinale*, it is urgently needed to produce more *D. officinale*. As we know, *D. officinale* originates from tropical and subtropical regions, with an optimum growth temperature range between 15 and 28 °C [38]. Thus, cold stress is one of the most destructive environmental factors limiting the planting and production of *D. officinale*, especially in northern China. Improvement of cold tolerance is the most efficient, rapid and economic approach for accelerating production of *D. officinale* [33,39].

The NAC transcription factor family is a plant-specific transcription factor family, which plays key roles in stress response and tolerance by regulating the expression of stress-related genes in plants [1,40]. Previous studies have demonstrated that NAC genes also have a great impact on plant cold tolerance and some key cold-related regulation pathways have been revealed, such as the NAC-DREB and NAC-CBF-COR signaling pathways [41]. However, NAC genes, especially those involved in cold tolerance in *D. officinale*, have not been well understood up to now. In this study, 110 NAC genes were identified in *D. officinale* through a genome-wide search method, which is similar to Arabidopsis (105), rice (137), poplar (163) and potato (110) [29]. Based on phylogenetic analysis and the classification criteria of Arabidopsis and rice, these 110 DoNACs can be further categorized into 15 subfamilies, and the potential functions of them were also pried by the homologs in Arabidopsis and rice. Sequence analysis found that the N-terminus of DoNACs was rather conserved while the C-terminus was highly variable. Furthermore, nine conserved protein motifs were predicted, of which, Motifs 1–8 were widely found in almost all of the DoNACs while Motif 9 was only present in a few DoNACs, adjacent to the C-terminus. It is interesting that DoNACs containing Motif 9 were completely clustered into independent evolutionary branches in the evolutionary tree and displayed high expression in root (such as *DoNAC29*) and stem (such as *DoNAC6*) tissues. Functional prediction found that Motif 9 was related to plant energy metabolism, speculating that it might be involved in regulating nutrient transport during root and stem development.

Based on RNA-seq data, a total of 31 DoNACs showed significantly differential expression between cold and control treatment, of which 11 were up-regulated and 20 were down-regulated. To verify the cold-responsive candidates, the expressions of five up-regulated and five down-regulated genes were further validated by the qRT-PCR method. There was very good consistancy between the results from the RNA-seq and qRT-PCR, demonstrating their role in cold response. The cold-responsive DoNAC102 was significantly induced to be 10 times up-regulated expression by cold stress, which belonged to the NAC22 subfamily and had rich ABRE, TGA-element and CGTCA-motif elements in its promoter [42]. It suggested that *DoNAC102* might mediate hormones to respond to cold stress. The cold-responsive *DoNAC56*, belonging to the ATAF subfamily, was also induced to up-regulated expression by cold stress. Previous studies have demonstrated that the ATAF subfamily generally play a vital role in abiotic stress tolerance. Furthermore, there were two LTR elements as well as many other stress-responsive elements in its promoter regions. These results suggested that *DoNAC56* might be a key candidate involved in cold tolerance in *D. officinale*. Additionally, some DoNACs were induced to down-regulated expression by cold, which might be considered a valuable target for gene editing study.

## 4. Materials and Methods

### 4.1. Genome-Wide Identification of NAC Family in D. officinale

The whole-genome reference and annotated protein sequences of the *D. officinale* genome were retrieved from the Herbal Medicine Omics Database (http://202.203.187.112/herbalplant/ (accessed on 15 March 2023)) and then used as the local protein database. NAC proteins in Arabidopsis and rice were downloaded from the TAIR (https://www.arabidopsis.org/ (accessed on 15 March 2023)) and Rice databases (http://rice.plantbiology.msu.edu/ (accessed on 15 March 2023)), respectively, to perform a BLASTP search against the local protein database with the threshold of E-value < 10^−5^. Furthermore, the Hidden Markov Model (HMM) profile of the NAC (PF01849) and NAM (PF02365) were downloaded from the PFAM database (http://pfam.xfam.org/ (accessed on 15 March 2023)) to search against the local protein database by HMMER 3.0 with the threshold of E-value < 10^−5^. The results from these two methods were integrated together and the redundant was manually removed to obtain the putative NAC proteins in *D. officinale* (DoNACs). To confirm the accuracy of prediction, these putative DoNACs were further submitted to the PFAM databases (https://pfam.xfam.org (accessed on 25 March 2023)) and SMART databases (https://smart.embl-heidelberg.de/ (accessed on 25 March 2023)) and only those with NAC or NAM domains remained as candidate DoNAC proteins. Finally, the EXPASy online software (https://www.expasy.org/ (accessed on 10 May 2023)) was used to predict the molecular weight (MW), length of amino acid (AA) and isoelectric point (pI) and their subcellular localization was predicted using the Plant-mPLoc subcellular location tool (http://www.csbio.sjtu.edu.cn/bioinf/plant-multi/ (accessed on 10 May 2023)).

### 4.2. Phylogenetic Relationship, Conserved Motif and Cis-Element Analysis

The protein sequences of the identified DoNACs, together with 105 AtNACs and 137 OsNACs, were used to perform a multiple sequence alignment based on the ClustalW tool and the phylogenetic tree was constructed using RAxML software with a bootstrap value of 1000 replications. The obtained tree was further edited and visualized using the Interactive Tree Of Life online tool (https://itol.embl.de/ (accessed on 10 May 2023)). The conserved regions were determined based on the alignment file. Meanwhile, the conserved protein motif was predicted using the MEME online tool (http://alternate.meme-suite.org/tools/meme (accessed on 10 May 2023)) with the maximum motif set to 10. Additionally, the upstream 1500 bp of the TSS (transcription start site) of the identified DoNACs were extracted as the putative promoter sequences, and the cis-acting elements were predicted using the PlantCARE online tool (https://bioinformatics.psb.ugent.be/webtools/plantcare/html/ (accessed on 10 May 2023)).

### 4.3. Expression Analysis in Diverse Tissues and under Cold Stress

RNA-seq datasets of 8 tissues, including column, sepal, root, root tip, stem, leaf, lip and flower buds, were downloaded from the NCBI sequence read archive (SRA) database with the accession No. PRJNA348403, and the RNA-seq data under cold stress and control conditions were also downloaded from the SRA database with the accession No. PRJNA314400 (Table S4). After quality control, the clean reads were mapped onto the reference sequences of *D. officinale* using the Hisat2 tool [43], and the transcripts per million (TPM) were calculated by StringTie v2.1.2 [44]. The differential expressed gene was detected by the DESeq2 tool with the cut-off parameter: FDR < 0.05 and |log2foldchange| > 1. The heatmap of the expression patterns was drawn by R software version 4.1.0.

### 4.4. qPCR Validation of the Cold-Responsive DoNACs

Plant materials were prepared following the method described previously [33] and the leaves were collected after 24 h of treatment under control and cold stress conditions with three to five plants mixed together. Three biological replications were performed. All samples were stored at −80 °C until RNA extraction using the RNA Easy Fast Plant Tissue Kit (Tiangen, Beijing, China)and 2 μg total RNA was used to synthesize the cDNA using Geneseed^®^ II First Strand cDNA Synthesis Kit (Geneseed, Guangzhou, China) according to the manufacturer’s protocol. A total of 10 cold-responsive DoNACs were randomly selected for validation by qRT-PCR analysis, including 5 up- and 5 down-regulated candidates, respectively. The qRT-PCR reaction was performed using the ABI 7500 instrument (ABI7500, ABI, Foster City, CA, USA) with Geneseed^®^ qPCR SYBR^®^ Green Master Mix (Geneseed, Guangzhou, China) with the primers listed in Table S5. The reaction mixture was 20 μL in volume, including 10 μL of Geneseed^®^ qPCR SYBR^®^ Green Master Mix, 0.5 μL of each primer (10 μM), 0.4 μL 50× ROX Reference Dye 2, 2 μL of the cDNA template and 7.6 μL of RNase free H_2_O. The thermal cycling program was as follows: 95 °C for 5 min, followed by 40 cycles at 95 °C for 10 s and 60 °C for 34 s. DoGAPDH was used as the internal reference gene. The expression levels of the targeted genes were calculated by the 2^−ΔΔCt^ method.

### 4.5. Subcellular Localization of DoNAC-GFP Fusion Proteins

To get further insights into the biological function of the cold-responsive DoNACs, 2 candidates were selected for validating their subcellular localization, including DoNAC39 and DoNAC58. They were firstly cloned to integrate into the pBI121-GFP vector. Then, the constructed recombined vector was separately transformed into Agrobacterium tumefaciens strain GV3101 and was further injected into 4-week-old tobacco leaves for transient expression of DoNACs. Subcellular localization of them was determined by the imaging of the leaves after 48 h of agro-infiltration using a laser confocal microscope (Olympus, Tokyo, Japan).

## 5. Conclusions

In this study, a total of 110 NAC genes belonging to 15 subfamilies were identified in *D. officinale* at the genome level. The genomic organization, phylogenetic relationship, conserved domain and cis-element of these DoNACs were systematically investigated. It is obvious that the genes with closer phylogenetic relationships shared more similar protein motifs and cis-element compositions. Furthermore, the tissue-specific and cold-responsive DoNACs were obtained based on RNA-seq data. Then, 10 cold-responsive DoNACs were further validated by qRT-PCR analysis, and the subcellular localization of two down-regulated candidates (DoNAC39 and DoNAC58) was also revealed to obtain some key cold-responsive candidates. This study provided the targets for further functional studies, which will contribute to the genetic improvement of cold tolerance in *D. officinale* and other herbs.

## Figures and Tables

**Table 1:** The NAC genes identified in *D. officinale*.

Gene ID	Gene Name	Protein Size (AA)	pI	Molecular Weight (kDa)	Subcellular
Dendrobium_GLEAN_10145674	DoNAC1	403	5.83	45.14922	Nuclear
Dendrobium_GLEAN_10132016	DoNAC2	214	8.96	24.05615	Nuclear
Dendrobium_GLEAN_10127752	DoNAC3	201	10.28	23.17154	Mitochondrial
Dendrobium_GLEAN_10125186	DoNAC4	341	6.88	39.36333	Nuclear
Dendrobium_GLEAN_10125242	DoNAC5	105	5	11.45904	Cytoplasmic
Dendrobium_GLEAN_10121576	DoNAC6	338	7.11	38.55624	Nuclear
Dendrobium_GLEAN_10119429	DoNAC7	313	6.55	36.06974	Nuclear
Dendrobium_GLEAN_10119030	DoNAC8	125	9.54	14.32841	Mitochondrial
Dendrobium_GLEAN_10116337	DoNAC9	280	5.81	32.51057	Cytoplasmic
Dendrobium_GLEAN_10115867	DoNAC10	347	4.33	39.12014	Nuclear
Dendrobium_GLEAN_10114825	DoNAC11	195	5.28	22.19483	Cytoplasmic
Dendrobium_GLEAN_10113712	DoNAC12	709	4.63	78.28001	Nuclear
Dendrobium_GLEAN_10113713	DoNAC13	1174	4.78	129.50276	Nuclear
Dendrobium_GLEAN_10110893	DoNAC14	297	9.1	34.35212	Nuclear
Dendrobium_GLEAN_10110543	DoNAC15	398	6.68	45.2573	Nuclear
Dendrobium_GLEAN_10110544	DoNAC16	119	6.29	14.24564	Cytoplasmic
Dendrobium_GLEAN_10110681	DoNAC17	341	6.32	38.85693	Nuclear
Dendrobium_GLEAN_10109120	DoNAC18	251	9.36	28.12291	Nuclear
Dendrobium_GLEAN_10106022	DoNAC19	117	9.68	13.13935	Extracellular
Dendrobium_GLEAN_10104194	DoNAC20	282	9.3	32.25563	Nuclear
Dendrobium_GLEAN_10100250	DoNAC21	300	5.37	34.18254	Nuclear
Dendrobium_GLEAN_10098876	DoNAC22	174	9	20.14462	Nuclear
Dendrobium_GLEAN_10096163	DoNAC23	641	8.75	72.99217	Nuclear
Dendrobium_GLEAN_10091520	DoNAC24	284	8.7	31.92515	Chloroplast
Dendrobium_GLEAN_10089166	DoNAC25	316	9.49	35.73608	Nuclear
Dendrobium_GLEAN_10089170	DoNAC26	318	5.75	36.07187	Cytoplasmic
Dendrobium_GLEAN_10088322	DoNAC27	130	4.62	15.0337	Nuclear
Dendrobium_GLEAN_10087230	DoNAC28	162	9.18	18.98751	Mitochondrial
Dendrobium_GLEAN_10083779	DoNAC29	293	8.37	32.75773	Nuclear
Dendrobium_GLEAN_10080940	DoNAC30	262	5.23	30.17884	Nuclear
Dendrobium_GLEAN_10080942	DoNAC31	262	5.32	30.16295	Cytoplasmic
Dendrobium_GLEAN_10079456	DoNAC32	285	5.95	32.94021	Nuclear
Dendrobium_GLEAN_10078554	DoNAC33	101	4.78	11.51279	Cytoplasmic
Dendrobium_GLEAN_10078555	DoNAC34	359	6.05	40.56424	Cytoplasmic
Dendrobium_GLEAN_10077726	DoNAC35	160	9.74	18.50435	Mitochondrial
Dendrobium_GLEAN_10073743	DoNAC36	661	6	75.60385	Nuclear
Dendrobium_GLEAN_10072577	DoNAC37	151	8.76	17.89539	Nuclear
Dendrobium_GLEAN_10069509	DoNAC38	208	8.57	24.32624	Cytoplasmic
Dendrobium_GLEAN_10069510	DoNAC39	267	5.08	31.15486	Nuclear
Dendrobium_GLEAN_10068907	DoNAC40	308	6.49	35.85838	Nuclear
Dendrobium_GLEAN_10066548	DoNAC41	304	7.69	34.92676	Nuclear
Dendrobium_GLEAN_10065340	DoNAC42	221	9.17	25.23475	Nuclear
Dendrobium_GLEAN_10061197	DoNAC43	128	8.28	14.26358	Cytoplasmic
Dendrobium_GLEAN_10061198	DoNAC44	220	8.59	24.31788	Extracellular
Dendrobium_GLEAN_10061199	DoNAC45	112	4.69	12.52021	Extracellular
Dendrobium_GLEAN_10060620	DoNAC46	251	8.78	28.10788	Nuclear
Dendrobium_GLEAN_10060621	DoNAC47	97	4.52	11.14347	Nuclear
Dendrobium_GLEAN_10058619	DoNAC48	278	8.77	31.99242	Cytoplasmic
Dendrobium_GLEAN_10053684	DoNAC49	313	8.92	35.57001	Cytoplasmic
Dendrobium_GLEAN_10053685	DoNAC50	187	8.48	20.87264	Nuclear
Dendrobium_GLEAN_10052939	DoNAC51	251	8.95	28.01581	Nuclear
Dendrobium_GLEAN_10052503	DoNAC52	311	9	34.53148	Nuclear
Dendrobium_GLEAN_10052536	DoNAC53	546	4.51	61.31671	Nuclear
Dendrobium_GLEAN_10051831	DoNAC54	430	6.2	47.6221	Nuclear
Dendrobium_GLEAN_10049252	DoNAC55	319	9.14	35.37876	Nuclear
Dendrobium_GLEAN_10049133	DoNAC56	630	8.22	71.57787	Cytoplasmic
Dendrobium_GLEAN_10048805	DoNAC57	378	8.38	41.8827	Nuclear
Dendrobium_GLEAN_10048260	DoNAC58	321	5.32	36.82507	Nuclear
Dendrobium_GLEAN_10046985	DoNAC59	160	9.32	18.69425	Nuclear
Dendrobium_GLEAN_10046111	DoNAC60	262	6.94	29.87379	Extracellular
Dendrobium_GLEAN_10045817	DoNAC61	236	4.94	27.23745	Nuclear
Dendrobium_GLEAN_10043622	DoNAC62	352	6.76	41.10419	Nuclear
Dendrobium_GLEAN_10042843	DoNAC63	345	7.6	39.17742	Nuclear
Dendrobium_GLEAN_10042799	DoNAC64	277	8.24	30.96703	Nuclear
Dendrobium_GLEAN_10042684	DoNAC65	369	6.01	41.49966	Nuclear
Dendrobium_GLEAN_10042333	DoNAC66	334	8.56	38.10982	Nuclear
Dendrobium_GLEAN_10042836	DoNAC67	437	6.26	50.02975	Nuclear
Dendrobium_GLEAN_10042279	DoNAC68	222	8.69	24.99947	Nuclear
Dendrobium_GLEAN_10042421	DoNAC69	157	9.64	18.32222	Cytoplasmic
Dendrobium_GLEAN_10041251	DoNAC70	286	7.63	33.8024	Nuclear
Dendrobium_GLEAN_10040136	DoNAC71	245	5.73	27.80819	Cytoplasmic
Dendrobium_GLEAN_10039845	DoNAC72	283	6.67	32.33944	Nuclear
Dendrobium_GLEAN_10039511	DoNAC73	409	6.26	45.40578	Nuclear
Dendrobium_GLEAN_10037990	DoNAC74	319	5.63	36.12287	Cytoplasmic
Dendrobium_GLEAN_10036377	DoNAC75	116	6.28	13.35206	Nuclear
Dendrobium_GLEAN_10036378	DoNAC76	335	8.37	36.74328	Nuclear
Dendrobium_GLEAN_10036159	DoNAC77	326	8.48	36.90069	Nuclear
Dendrobium_GLEAN_10034487	DoNAC78	146	7.76	17.00228	Cytoplasmic
Dendrobium_GLEAN_10032744	DoNAC79	309	8.62	35.55115	Cytoplasmic
Dendrobium_GLEAN_10032445	DoNAC80	379	9.07	43.11576	Nuclear
Dendrobium_GLEAN_10030109	DoNAC81	291	9.05	32.6348	Nuclear
PEQU_23244-D2	DoNAC82	345	6.09	39.06895	Nuclear
Dendrobium_GLEAN_10030254	DoNAC83	342	5.79	38.68952	Nuclear
Dendrobium_GLEAN_10027918	DoNAC84	534	9.16	60.5709	Nuclear
Dendrobium_GLEAN_10027115	DoNAC85	555	8.97	64.00039	Plasma Membrane
Dendrobium_GLEAN_10026921	DoNAC86	283	6.67	32.33944	Nuclear
Dendrobium_GLEAN_10023733	DoNAC87	593	5.02	66.27489	Nuclear
Dendrobium_GLEAN_10022335	DoNAC88	371	5.27	41.05842	Cytoplasmic
Dendrobium_GLEAN_10018741	DoNAC89	198	6.16	22.51354	Nuclear
Dendrobium_GLEAN_10016232	DoNAC90	330	6.8	37.84653	Nuclear
Dendrobium_GLEAN_10016023	DoNAC91	298	6.08	33.85566	Nuclear
Dendrobium_GLEAN_10015903	DoNAC92	199	5.81	22.27767	Nuclear
Dendrobium_GLEAN_10014831	DoNAC93	130	4.19	14.78027	Nuclear
Dendrobium_GLEAN_10014832	DoNAC94	561	5.74	64.18911	Nuclear
Dendrobium_GLEAN_10014534	DoNAC95	340	5.96	38.01689	Nuclear
Dendrobium_GLEAN_10014535	DoNAC96	183	5.89	20.71126	Cytoplasmic
Dendrobium_GLEAN_10014195	DoNAC97	109	5.35	12.24524	Cytoplasmic
Dendrobium_GLEAN_10013786	DoNAC98	177	9.6	19.70194	Chloroplast
Dendrobium_GLEAN_10010728	DoNAC99	313	7.8	35.8342	Nuclear
Dendrobium_GLEAN_10010634	DoNAC100	148	8.49	17.43163	Nuclear
Dendrobium_GLEAN_10010200	DoNAC101	103	9.94	12.06804	Mitochondrial
Dendrobium_GLEAN_10008910	DoNAC102	321	6.06	36.54962	Nuclear
Dendrobium_GLEAN_10008576	DoNAC103	200	9.05	23.83835	Plasma Membrane
Dendrobium_GLEAN_10007482	DoNAC104	311	5.68	35.97639	Nuclear
Dendrobium_GLEAN_10006697	DoNAC105	178	9.43	21.16647	Cytoplasmic
Dendrobium_GLEAN_10005041	DoNAC106	230	9.61	26.40513	Nuclear
Dendrobium_GLEAN_10003671	DoNAC107	144	9.46	17.19184	Cytoplasmic
Dendrobium_GLEAN_10003465	DoNAC108	275	8.66	31.21719	Nuclear
Dendrobium_GLEAN_10003618	DoNAC109	148	8.39	17.4567	Nuclear
Dendrobium_GLEAN_10000578	DoNAC110	131	7.87	14.95097	Nuclear

## Data Availability

All of the datasets supporting the results of this article are included within the article and its additional files.

## Supplementary Materials

The following supporting information can be downloaded at: https://www.mdpi.com/article/10.3390/plants12203626/s1, Table S1: The cis-elements identified in the promoter regions of the 110 DoNAC genes; Table S2: The FPKM value of each DoNAC gene in different organs; Table S3: The FPKM value of each DoNAC gene in different organs; Table S4: The information of the RNA-seq data used in this study; Table S5: Primers used for qPCR analysis in this study.
